# Immunohistochemical and Transcriptomic Assessment of Cytochrome 2J2 in Prostate and Renal Cancer

**DOI:** 10.3390/cancers18172809

**Published:** 2026-08-29

**Authors:** Yousef M. Al-saraireh, Fatemah OFO Alshammari, Awad Dmour, Ahmed. A. Al-abadleh, Mohannad Ja’Awin, Marwan Herzallah, Fadi Sawaqed, Anas O. Satari, Sameeh A. Al-sarayreh, Sa’ed M. Al-dalain, Aiman Al-Qtaitat, Jehad M. Al Shuneigat, Abulmaaty M. Elsayed, Mohammad Salem Hareedy

**Affiliations:** 1 Department of Pharmacology, Faculty of Medicine, University of Mutah, P.O. Box 7, Al-Karak 61710, Jordan; gregorio@mutah.edu.jo; 2 Department of Medical Laboratory Technology, Faculty of Health Sciences, The Public Authority for Applied Education and Training, Kuwait City 15432, Kuwait; fo.alowayed@paaet.edu.kw; 3 Department of Orthopedics and Traumatology, Faculty of Medicine, “Grigore T. Popa” University of Medicine and Pharmacy, 700115 Iasi, Romania; dmour-awad@umfiasi.ro; 4 Department of Urology, Faculty of Medicine, MUST University, Cairo 12568, Egypt; 25210057@arab-board.education; 5 Department of Special Surgery, Faculty of Medicine, University of Mutah, P.O. Box 7, Al-Karak 61710, Jordan; 420201503769@mutah.edu (M.J.); 120201503027@mutah.edu.jo (M.H.); fadi.sawaqed@mutah.edu.jo (F.S.); 6 Department of General Pediatrics, Maternity and Children’s Hospital at Al Bashir Hospital, Amman 11151, Jordan; satari@mutah.edu.jo; 7 Department of Biochemistry and Molecular Biology, Faculty of Medicine, University of Mutah, P.O. Box 7, Al-Karak 61710, Jordan; sameeh_sarayreh@mutah.edu.jo (S.A.A.-s.); dr.jehad@mutah.edu.jo (J.M.A.S.); 8 Department of Anatomy and Histology, Faculty of Medicine, University of Mutah, P.O. Box 7, Al-Karak 61710, Jordan; aimanaq@mutah.edu.jo (A.A.-Q.); abulsala@mutah.edu.jo (A.M.E.)

**Keywords:** cytochromes, cytochrome 2J2, immunohistochemistry, prostate cancer, renal cancer, transcriptomics

## Abstract

**Background:** Cytochrome P450 2J2 (CYP2J2) has been implicated in tumor biology, but its expression pattern and clinical significance in prostate and renal cancers remain poorly characterized. **Methods:** Commercially available tissue microarrays containing 208 renal and 121 prostate tissue specimens were used for immunohistochemical (IHC) characterization of CYP2J2 protein expression. We performed transcriptomic analysis and examined correlations with clinicopathological features and survival using the OncoDB 2.0 platform. **Results:** CYP2J2 immunoreactivity was mainly cytoplasmic, with minimal protein expression in both cancers. We identified positive staining in 7/100 (7.0%) prostate adenocarcinomas and 2/192 (1.0%) clear cell renal cell carcinoma (ccRCC) specimens, with negative expression in corresponding normal tissues. We detected no significant associations between protein expression and clinicopathological features in either cohort (all *p* > 0.05). In contrast, transcriptomic analysis revealed elevated CYP2J2 mRNA expression in both malignancies, with a slight increase in prostate adenocarcinoma (log_2_ fold change = 1.10) and marked overexpression in ccRCC (log_2_ fold change = 5.24) compared with normal tissues. We observed significant associations between CYP2J2 mRNA expression and pathological T stage in prostate adenocarcinoma (*p* = 0.0019) and pathological M stage in ccRCC (*p* = 0.0079). Moreover, higher CYP2J2 mRNA expression was associated with longer overall survival in ccRCC (HR = 0.64, 95% CI: 0.47–0.86; log-rank *p* = 0.0029) but not in prostate adenocarcinoma; however, these findings remain exploratory and do not establish independent prognostic value. **Conclusions:** Despite elevated transcript expression, CYP2J2 protein expression was infrequent in prostate adenocarcinoma and ccRCC, suggesting limited clinicopathological relevance and warranting further investigation using functional techniques.

## Simple Summary

Prostate cancer and kidney cancer are common cancers that require a better understanding of their disease-related molecular changes. Cytochrome P450 2J2 is an enzyme involved in fatty acid metabolism, but its role in these cancers remains unclear. This study examined Cytochrome P450 2J2 protein expression in tissues from prostate adenocarcinoma and clear cell renal cell carcinoma using immunohistochemistry. It evaluated messenger RNA expression and clinical associations using publicly available transcriptomic data. Protein staining was infrequent and weak in both tumor types, whereas messenger RNA expression was increased, particularly in clear cell renal cell carcinoma. Expression patterns varied with disease stage, and higher expression was associated with longer overall survival in clear cell renal cell carcinoma. Given that the protein and transcript data came from different cohorts, these findings should be considered exploratory. Further studies using matched and independent samples are necessary to clarify the clinical significance of Cytochrome P450 2J2.

## 1. Introduction

Prostate cancer and renal cell carcinoma (RCC) are among the most common urological cancers and continue to pose a significant global health burden. According to recent GLOBOCAN estimates, prostate cancer accounts for over 1.5 million new cases and over 420,000 deaths globally, making it the second most commonly diagnosed cancer and the fourth leading cause of cancer-related death in males. Meanwhile, kidney cancer causes about 443,000 new cases and 145,000 fatalities each year, with clear cell renal cell carcinoma (ccRCC) comprising about 75% of cases [1]. Despite significant treatment advancements in urological cancers—including radical prostatectomy, radiotherapy, androgen receptor pathway inhibitors, chemotherapy, immune checkpoint inhibitors, and tyrosine kinase inhibitors—disease recurrence, therapeutic resistance, and metastatic spread of disease remain the leading causes of death in both cancers [2,3]. Thus, there is an urgent need to identify novel biomarkers to improve diagnostic options, prognostic prediction, and therapy selection.

Cytochrome P450 (CYP) enzymes are monooxygenases that metabolize xenobiotics and anticancer drugs in addition to endogenous substrates such as steroid hormones, fatty acids, eicosanoids, and vitamins [4,5,6,7]. Importantly, CYP dysregulation contributes to many cancer hallmarks through its involvement in carcinogen activation, oxidative stress regulation, hormone metabolism, angiogenesis, inflammation, and drug resistance. Multiple CYP enzymes show abnormal expression in various human tumors and have been explored as diagnostic biomarkers and potential therapeutic targets [4,8,9,10].

Among CYP epoxygenases, Cytochrome P450 2J2 (CYP2J2) has attracted growing interest because it metabolizes arachidonic acid into epoxyeicosatrienoic acids (EETs). These lipid mediators participate in processes such as vascular homeostasis, inflammation, cellular apoptosis, and proliferation. Abnormal CYP2J2 expression is associated with the progression of several malignancies, and experimental studies have suggested tumor-promoting effects in selected cancer models. However, emerging evidence suggests that CYP2J2′s biological significance depends highly on tumor type and cellular context rather than representing a universally oncogenic factor. Differences in CYP2J2 expression among tumor types may reflect distinct tissue environments, metabolic states, cellular differentiation, or regulatory mechanisms [11,12,13,14,15,16,17]. Thus, characterizing CYP2J2 in individual tumor types is necessary before assigning a uniform tumor-promoting role to this enzyme.

While interest in CYP2J2 as a potential tumor-associated enzyme is growing, its expression pattern and clinical implications in prostate adenocarcinoma and ccRCC remain poorly understood. Previous studies have examined CYP2J2 using individual approaches—immunohistochemistry, molecular assays, or large-scale bioinformatic analyses—each providing partial and methodologically distinct insights. Earlier immunohistochemical studies demonstrated CYP2J2 protein distribution across human tissues and malignancies but were limited by small cohorts and a lack of comprehensive clinicopathological correlation in prostate adenocarcinoma and ccRCC [11,15,18]. Concurrently, database-based analyses have provided broader transcriptomic and clinical associations, particularly in ccRCC, but lacked tissue-level protein localization or validation in independent pathological cohorts [19]. In contrast, this is the first study to assess CYP2J2 protein expression by immunohistochemistry in both tumor types alongside transcriptomic profiling, clinicopathological analyses, and survival evaluation using publicly available datasets. We used these complementary approaches to characterize CYP2J2 at the protein and transcript levels, evaluate their respective associations with clinicopathological features and patient outcomes, and assess its potential clinical relevance in these two common urological cancers. Importantly, the immunohistochemical and transcriptomic datasets come from different patient populations and therefore do not provide matched patient-level validation.

## 2. Materials and Methods

### 2.1. Tissue Specimens and Immunohistochemistry

Two commercially available tissue microarrays (TMAs) from TissueArray.com LLC (Derwood, MD, USA) were used to assess formalin-fixed, paraffin-embedded (FFPE) tissue specimens. The prostate TMA (PR121) contained a single core for 121 tissue samples: 100 prostate adenocarcinomas, 3 leiomyosarcomas, 7 cases of simple hyperplasia, and 11 normal prostate tissues. The accompanying clinicopathological data included patient age, pathological diagnosis, tumor–node–metastasis (TNM) classification, American Joint Committee on Cancer (AJCC, 8th edition) clinical stage, and Gleason grade. The renal TMA (KD20810) contained 208 tissue cores: 192 ccRCC cases and 16 normal kidney tissues, with each case represented by one core. The clinicopathological data for the renal cohort included gender, age, pathological diagnosis, pathological T stage, lymph node involvement, distant metastasis, pathological stage, histological grade, and tumor size. The study was authorized by the Mutah University Faculty of Medicine’s Institutional Review and Ethics Committee and was conducted in compliance with the Declaration of Helsinki (Reference No. EC6-2326, dated 2 March 2026). Patient consent was not required because the Mutah University Faculty of Medicine’s Institutional Review and Ethics Committee granted an exemption (Reference No. EC6-2326, dated 2 March 2026), as the study used de-identified, commercially obtained TMAs.

TMA cores were initially de-waxed in xylene and rehydrated through serial graded ethanol solutions to distilled water. We performed heat-induced antigen retrieval using sodium citrate buffer (10 mM, pH 6.0) in a microwave oven (600 W) for 20 min. To block endogenous peroxidase activity, TMA cores were quenched with 5% hydrogen peroxide for 5 min, followed by triplicate washes in phosphate-buffered saline (PBS). TMA cores were then incubated with 5% normal goat serum for 20 min to reduce non-specific antibody binding. After that, we applied the primary anti-CYP2J2 antibody (MBS822016, MyBioSource, San Diego, CA, USA) to sections for 1 h at room temperature. After several PBS washes, tissue sections were incubated with reagents of the ImmPRESS^®^ HRP polymer detection system (Vector Laboratories, Burlingame, CA, USA) for 30 min. We then developed immunoreactivity by incubating TMA sections with 3,3′-diaminobenzidine (DAB, Vector Laboratories, Burlingame, CA, USA) for 5 min. TMA cores were then counterstained with hematoxylin, dehydrated, and mounted with coverslips. To assess the specificity of the CYP2J2 antibody, multiple control approaches were incorporated. We used prostate cancer, ccRCC, and bladder cancer tissues as positive controls; corresponding sections treated with the CYP2J2 primary antibody pre-incubated with the manufacturer-specified CYP2J2 blocking peptide (MBS823102, MyBioSource, USA) served as peptide-neutralization controls. Additionally, negative controls were processed by omitting the primary antibody. The positive-control tissues demonstrated the expected cytoplasmic immunoreactivity, whereas peptide-neutralized and primary-antibody-omission controls showed no or minimal staining. These controls confirmed the specificity of the immunohistochemical staining procedure in our present experimental setting.

### 2.2. Immunohistochemical Scoring

Two experienced pathologists, blinded to all clinicopathological data, independently examined CYP2J2 immunostaining. CYP2J2 immunoreactivity was predominantly cytoplasmic and was semi-quantitatively evaluated using the Allred scoring system [20,21]. This system yields a score that is the sum of two components. The first component is the percentage of cells showing positive staining, ranging from 0 to 5 (0 = none; 1 = <1%; 2 = 1–10%; 3 = 11–33%; 4 = 34–66%; 5 = >66%). The second component is the intensity score, which is assigned a grade of 0 (no staining), 1 (weak staining), 2 (moderate staining), or 3 (strong staining) based on the average staining intensity in tumor cells. The sum of these two scores yields the final Allred score, which ranges from 0 to 8. Scores of 0 to 2 denote negative expression, while scores of 3 to 8 indicate positive expression. We selected the Allred score ≥ 3 as an exploratory classification threshold based on the established use of the Allred scoring system in immunohistochemical biomarker studies. However, this cutoff has not been specifically validated for CYP2J2 expression in prostate adenocarcinoma or ccRCC. Therefore, the positive/negative categorization was used primarily to facilitate descriptive and clinicopathological analyses rather than as a disease-specific validated biomarker threshold. Moreover, the two observers jointly reviewed discrepancies to reach a consensus score. Because the final analysis was based on consensus scores, a formal interobserver agreement statistic was not calculated.

### 2.3. Pattern of CYP2J2 mRNA Expression and Associations with Clinicopathological Features and Survival

We used the OncoDB 2.0 web tool (https://oncodb.org, accessed on 3 July 2026) to assess CYP2J2 mRNA expression [22]. For prostate cancer, transcriptomic data from The Cancer Genome Atlas Prostate Adenocarcinoma (TCGA-PRAD) cohort were examined to assess CYP2J2 mRNA expression in tumor (505 cases) and normal prostate tissues (52 cases). For renal cancer, transcriptomic data from The Cancer Genome Atlas Kidney Renal Clear Cell Carcinoma (TCGA-KIRC) cohort were evaluated to assess CYP2J2 mRNA expression between ccRCC (545 cases) and normal kidney tissues (72 cases). RNA expression values in OncoDB are derived from TCGA RNA-seq data and normalized using the transcripts per million (TPM) method [22]. The expression values displayed by OncoDB were therefore interpreted as TPM-based normalized expression values.

We examined differences in CYP2J2 mRNA expression between tumor and normal tissues using the OncoDB expression analysis module. We also assessed associations between CYP2J2 mRNA expression and available clinicopathological variables using the OncoDB platform. For survival analysis, patients were divided into high- and low-CYP2J2 expression cohorts using a 50% expression cutoff in the OncoDB 2.0 survival analysis module. Patients with CYP2J2 gene expression levels above the 50% threshold were classified into the high-expression cohort, whereas patients with expression levels below the threshold were classified into the low-expression cohort. Overall survival was evaluated using Kaplan–Meier analysis, and the survival curves compared using the log-rank test. Hazard ratios (HRs) and 95% confidence intervals (CIs) reported by OncoDB were also recorded. Because the survival analysis was based on unadjusted expression-group comparisons, we did not perform a multivariable Cox regression model incorporating pathological stage, age, or other clinicopathological variables. Therefore, the observed survival associations should be interpreted as unadjusted associations rather than independent prognostic effects. Statistical significance was defined as a log-rank *p*-value <0.05.

### 2.4. Statistical Analysis

IBM SPSS Statistics version 29.0 (IBM Corp., Armonk, NY, USA) was used for immunohistochemical data analysis. CYP2J2 expression was classified as negative or positive based on the Allred score (Section 2.2). Associations with clinicopathological variables were assessed using the Pearson Chi-square test or Fisher’s exact test when expected cell counts were small due to the low frequency of CYP2J2-positive cases. Transcriptomic analyses were performed using the OncoDB 2.0 platform (TCGA-PRAD and TCGA-KIRC datasets), which provides built-in statistical comparisons between tumor and normal tissues and across clinicopathological variables. Results were reported as provided by the platform (TPM-normalized expression; Section 2.3). Overall survival was analyzed using Kaplan–Meier curves with the log-rank test to compare high- and low-expression groups (median cutoff). Hazard ratios and 95% confidence intervals were obtained from OncoDB. All survival analyses were unadjusted and exploratory. A two-sided *p* < 0.05 was considered statistically significant. Given the exploratory nature of the study and multiple comparisons, we interpreted the results cautiously.

## 3. Results

### 3.1. Clinicopathological Characteristics of the Study Cohorts

#### 3.1.1. Prostate Cohort

The primary prostate cancer cohort comprised 100 prostate adenocarcinoma specimens. The TMA also contained 3 leiomyosarcoma, 7 simple hyperplasia, and 11 normal prostate tissue specimens, which we analyzed separately as non-adenocarcinoma tissue types (Table 1). Among the 100 prostate adenocarcinoma cases, 35 (35.0%) were aged ≤ 65 years and 65 (65.0%) were aged > 65 years. The most common tumor depth was T2 (70/100, 70.0%), followed by T3 (26/100, 26.0%) and T4 (4/100, 4.0%). Lymph node metastasis occurred in 15 cases (15.0%), whereas distant metastasis occurred in 6 cases (6.0%). The majority of tumors were Stage II (51/100, 51.0%), followed by Stage III (34/100, 34.0%) and Stage IV (15/100, 15.0%). Regarding Gleason grade, Grade 3 was the most frequent (24/100, 24.0%), followed by Grade 5 (23/100, 23.0%), Grade 2 (19/100, 19.0%), Grade 1 (20/100, 20.0%), and Grade 4 (14/100, 14.0%).

#### 3.1.2. Renal Clear Cell Carcinoma Cohort

The renal cohort contained 208 kidney tissue specimens: 16 normal renal tissues (7.7%) and 192 ccRCC specimens (92.3%) (Table 2). We restricted the primary renal analysis to the 192 ccRCC cases and analyzed the 16 normal kidney tissues separately as controls. Among the ccRCC cases, 142 (74.0%) patients were aged < 65 years and 50 (26.0%) were aged ≥ 65 years; 132 (68.8%) were male, and 60 (31.3%) were female. T1 tumors comprised the majority of cases (133/192, 69.3%), followed by T2 tumors (53/192, 27.6%); T3 and T4 tumors were uncommon (5/192, 2.6% and 1/192, 0.5%, respectively). Lymph node metastasis occurred in 1 case (0.5%), and distant metastasis occurred in 1 case (0.5%). Most cases were Stage I (134/192, 69.8%), followed by Stage II (52/192, 27.1%); Stages III and IV accounted for 5 (2.6%) and 1 (0.5%) cases, respectively. Histological Grade 1 tumors predominated (173/192, 90.1%), followed by Grade 2 (18/192, 9.4%) and Grade 3 (1/192, 0.5%).

### 3.2. CYP2J2 Protein Expression

**Figure 1:**
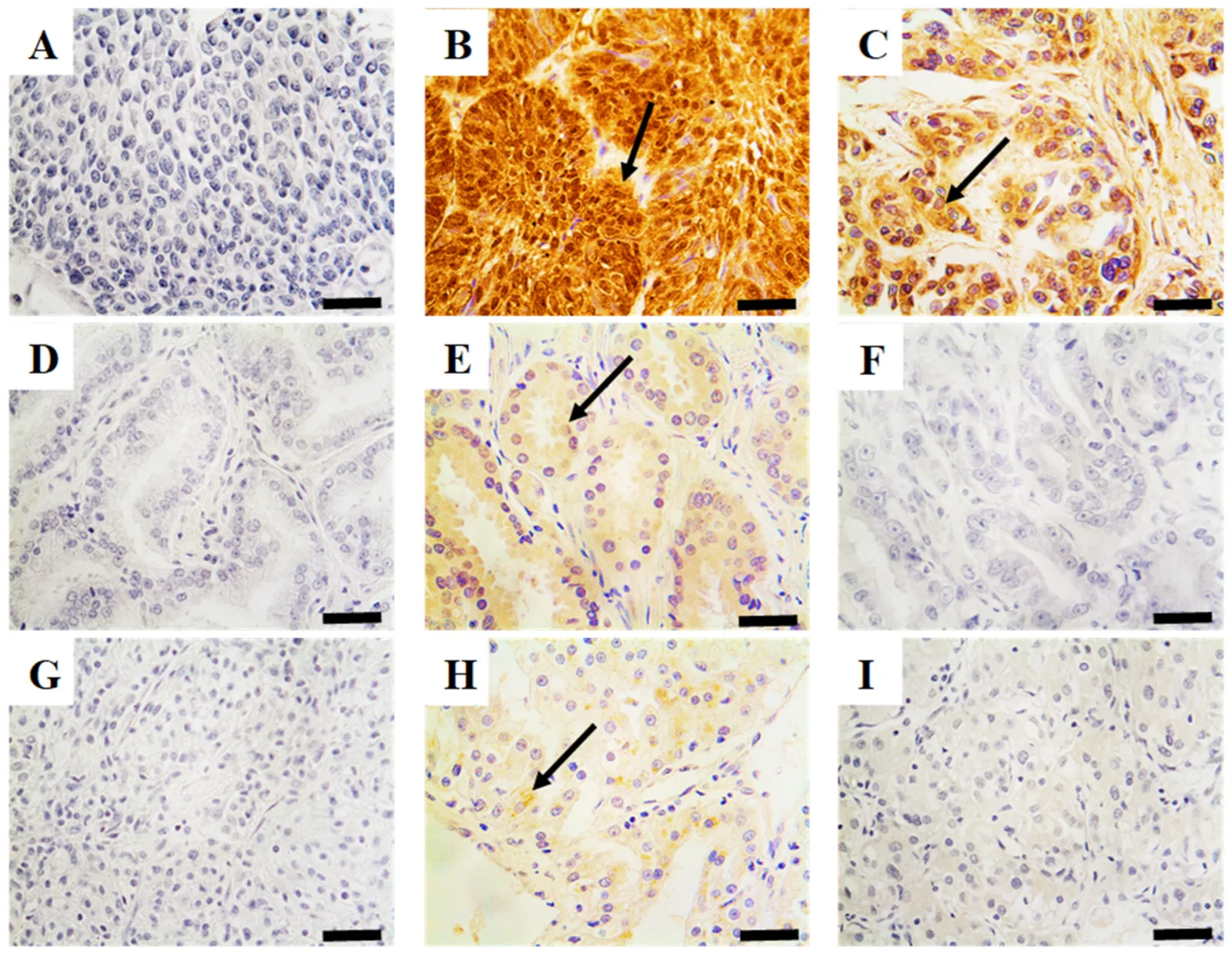
CYP2J2 expression in different experimental controls. (**A**) No CYP2J2 immunoreactivity was displayed in bladder cancer negative control (omission of primary antibody); (**B**) strong CYP2J2 immunoreactivity was exhibited in bladder cancer tissue; (**C**) minimal CYP2J2 immunoreactivity was displayed in bladder cancer tissue treated with neutralized primary antibody; (**D**) no CYP2J2 immunoreactivity was seen in prostate cancer negative control (omission of primary antibody); (**E**) CYP2J2 immunoreactivity was exhibited in prostate cancer positive control; (**F**) no CYP2J2 immunoreactivity was displayed in prostate cancer tissue treated with neutralized primary antibody; (**G**) no CYP2J2 immunoreactivity was seen in ccRCC negative control (omission of primary antibody); (**H**) CYP2J2 immunoreactivity was exhibited in ccRCC positive control; (**I**) no CYP2J2 immunoreactivity was displayed in ccRCC tissue treated with neutralized primary antibody. Arrows indicate representative CYP 2J2-positive cells. All images were captured at ×400 magnification. Scale bar = 50 μm.

Immunohistochemical analysis revealed CYP2J2 positivity predominantly in the cytoplasm of epithelial tumor cells in both prostate and kidney tissues. We validated the specificity of CYP2J2 staining using suitable positive and negative controls. The positive control, bladder cancer tissue, showed strong cytoplasmic immunoreactivity. Conversely, negative controls prepared by omitting the primary antibody or using a peptide-neutralized primary antibody showed no or minimal immunoreactivity, confirming the specificity of the immunohistochemical procedure (Figure 1).

#### 3.2.1. CYP2J2 Protein Expression in Prostate Specimens

**Figure 2:**
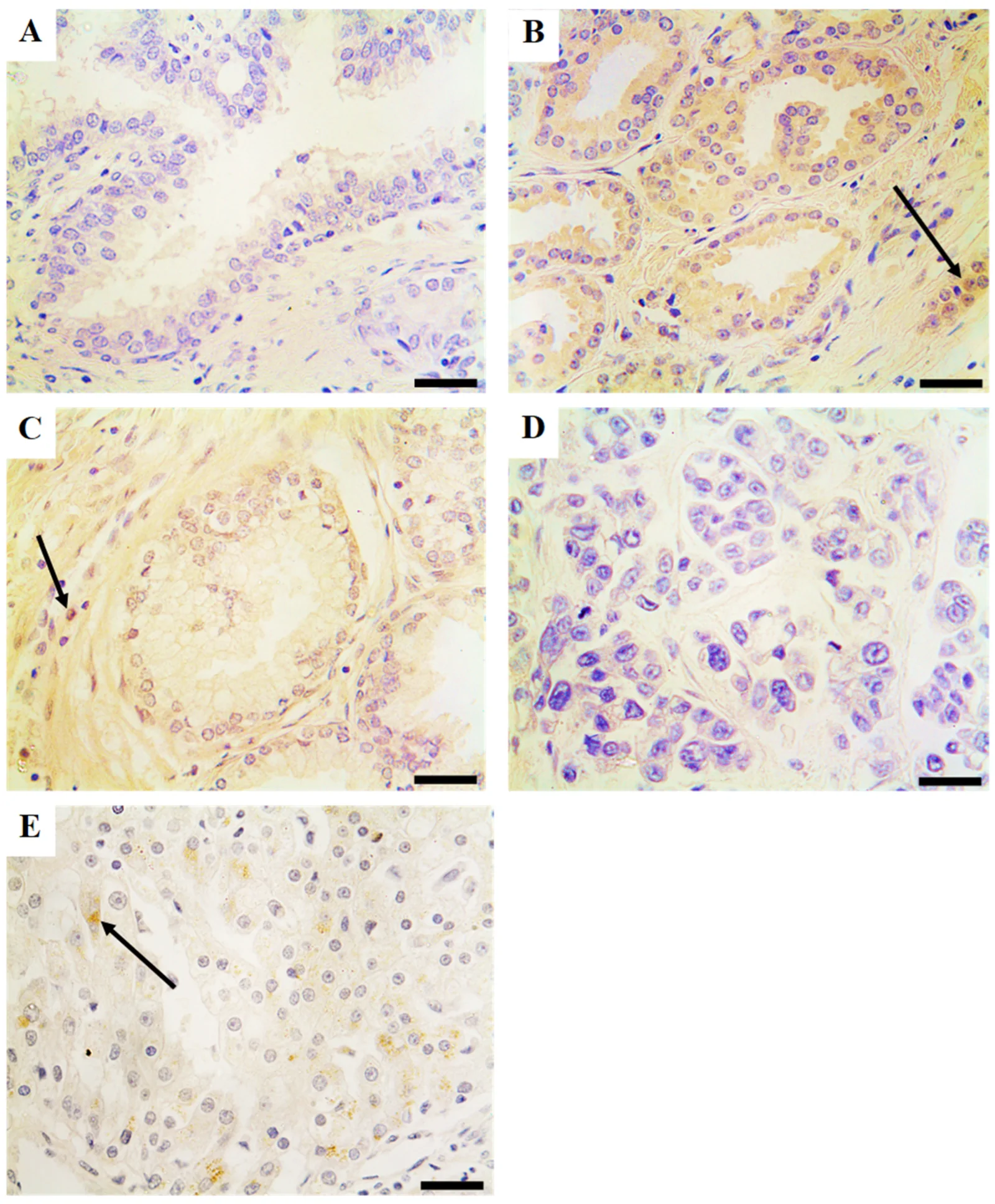
CYP2J2 expression in prostate and renal cancer. (**A**) No CYP2J2 immunoreactivity was displayed in normal prostate tissue; (**B**) CYP2J2 immunoreactivity was exhibited in prostate adenocarcinoma tissue; (**C**) CYP2J2 immunoreactivity was exhibited in prostate hyperplasia tissue; (**D**) no CYP2J2 immunoreactivity was displayed in normal kidney tissue; (**E**) CYP2J2 immunoreactivity was exhibited in ccRCC tissue. Arrows indicate representative CYP 2J2-positive cells. All images were captured at ×400 magnification. Scale bar = 50 μm.

Among the 100 prostate adenocarcinoma specimens, CYP2J2-positive immunoreactivity was observed in 7 cases (7.0%), while 93 cases (93.0%) were negative. (Table 1, Figure 2). For completeness, CYP2J2 immunoreactivity was observed in 2/7 (28.6%) simple hyperplasia specimens, whereas all three leiomyosarcoma specimens and all 11 normal prostate specimens were negative. Because of the very small sample size, we considered these findings descriptive and did not include them in the primary statistical analysis. However, CYP2J2 expression showed no statistically significant association with age (*p* = 0.236), depth of invasion (*p* = 0.239), lymph node metastasis (*p* = 1.000), distant metastasis (*p* = 0.361), clinical stage (*p* = 0.869), or Gleason grade (*p* = 0.078). Given that only 7 of 100 prostate specimens (7.0%) were CYP2J2-positive, these analyses had limited statistical power; therefore, the absence of statistically significant associations does not indicate that no associations exist.

#### 3.2.2. CYP2J2 Protein Expression in ccRCC

Only two of the 208 renal specimens (1.0%) showed positive CYP2J2 immunoreactivity, whereas 206 (99.0%) were negative. Both positive cases occurred among the 192 ccRCC specimens, while all 16 normal kidney tissues were negative. Among the 192 ccRCC cases, we observed no statistically significant associations between CYP2J2 protein expression and sex (*p* = 0.528), age (*p* = 1.000), T stage (*p* = 0.521), lymph node metastasis (*p* = 1.000), distant metastasis (*p* = 1.000), clinical stage (*p* = 0.514), or histological grade (*p* = 0.189). Because only 2 of 192 ccRCC cases were CYP2J2-positive, these analyses had extremely limited statistical power. Therefore, the non-significant findings reflect an inability to reliably detect associations rather than evidence that such associations are absent.

### 3.3. CYP2J2 Gene Expression

#### 3.3.1. CYP2J2 Gene Expression in Prostate Adenocarcinoma

**Figure 3:**
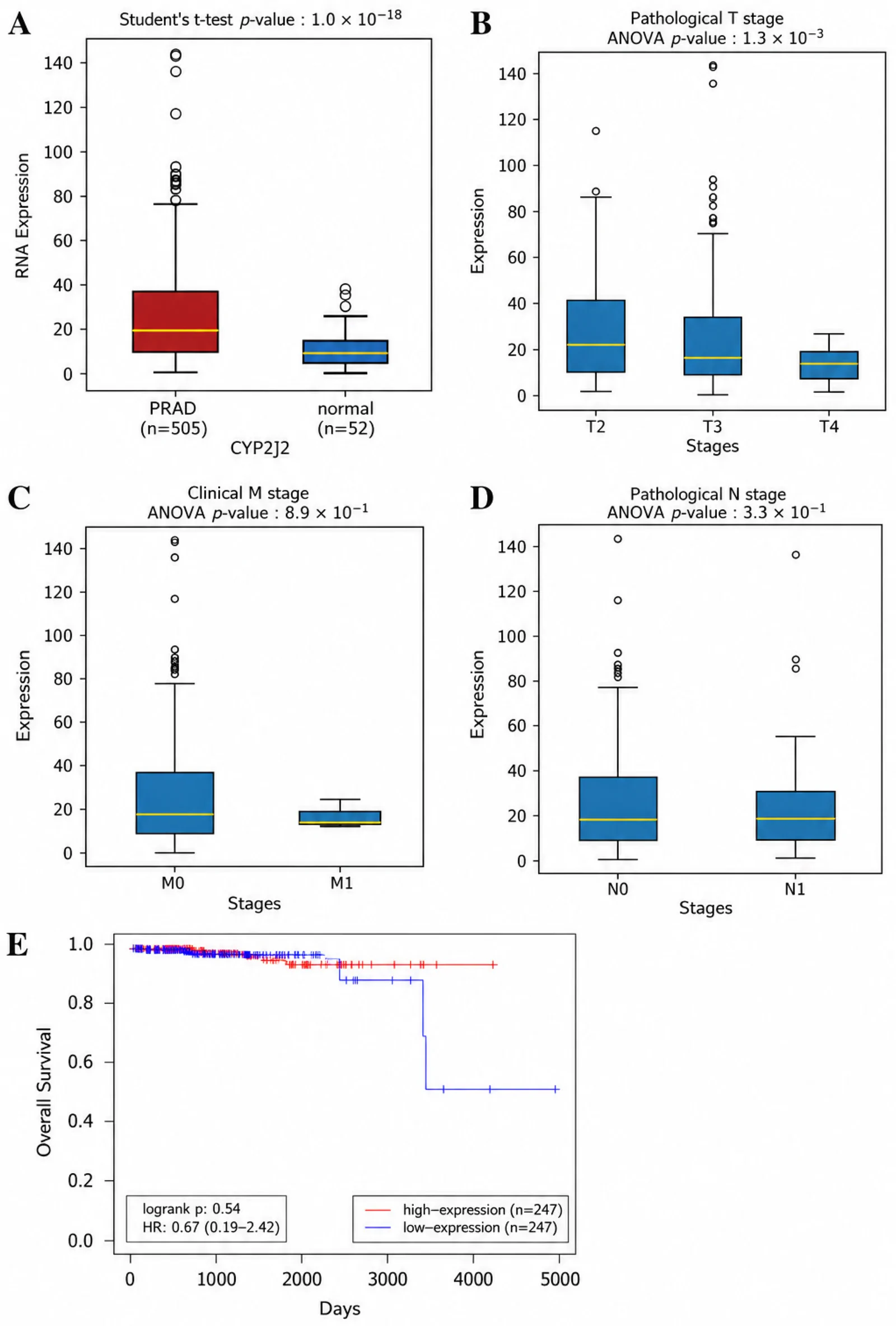
Transcriptomic analysis of CYP2J2 mRNA expression in prostate adenocarcinoma using the TCGA-PRAD cohort. (**A**) Boxplot comparing CYP2J2 mRNA expression between prostate adenocarcinoma (PRAD; *n* = 505) and normal prostate tissues (*n* = 52). (**B**) Association between CYP2J2 mRNA expression and pathological T stage. (**C**) Association between CYP2J2 mRNA expression and pathological M stage. (**D**) Association between CYP2J2 mRNA expression and pathological N stage. (**E**) Kaplan–Meier overall survival analysis according to CYP2J2 mRNA expression.

Transcriptomic analysis revealed slightly elevated CYP2J2 mRNA levels in prostate cancer compared with normal prostate tissue (Figure 3). The average expression level was 25.6 in tumor samples versus 10.4 in normal tissues (log2 fold change = 1.10), indicating mild transcriptional overexpression. Importantly, CYP2J2 mRNA expression was significantly associated with pathological T stage (*p* = 0.0019); however, the pattern was non-linear, with the highest mean expression in T2 tumors (62.4), followed by T3 tumors (54.3), and the lowest in T4 tumors (37.8). Thus, CYP2J2 expression did not progressively increase with advancing pathological T stage; rather, expression decreased at the most advanced T stage. Conversely, we detected no significant associations with pathological N stage (*p* = 0.33) or pathological M stage (*p* = 0.89). Moreover, CYP2J2 transcript levels were not significantly associated with overall survival in the prostate adenocarcinoma cohort according to Kaplan–Meier analysis (log-rank *p* = 0.54; HR = 0.67, 95% CI: 0.19–2.42). Because this analysis was based on an unadjusted comparison of expression-defined groups, it does not establish whether CYP2J2 expression is an independent prognostic factor.

#### 3.3.2. CYP2J2 Gene Expression in ccRCC

**Figure 4:**
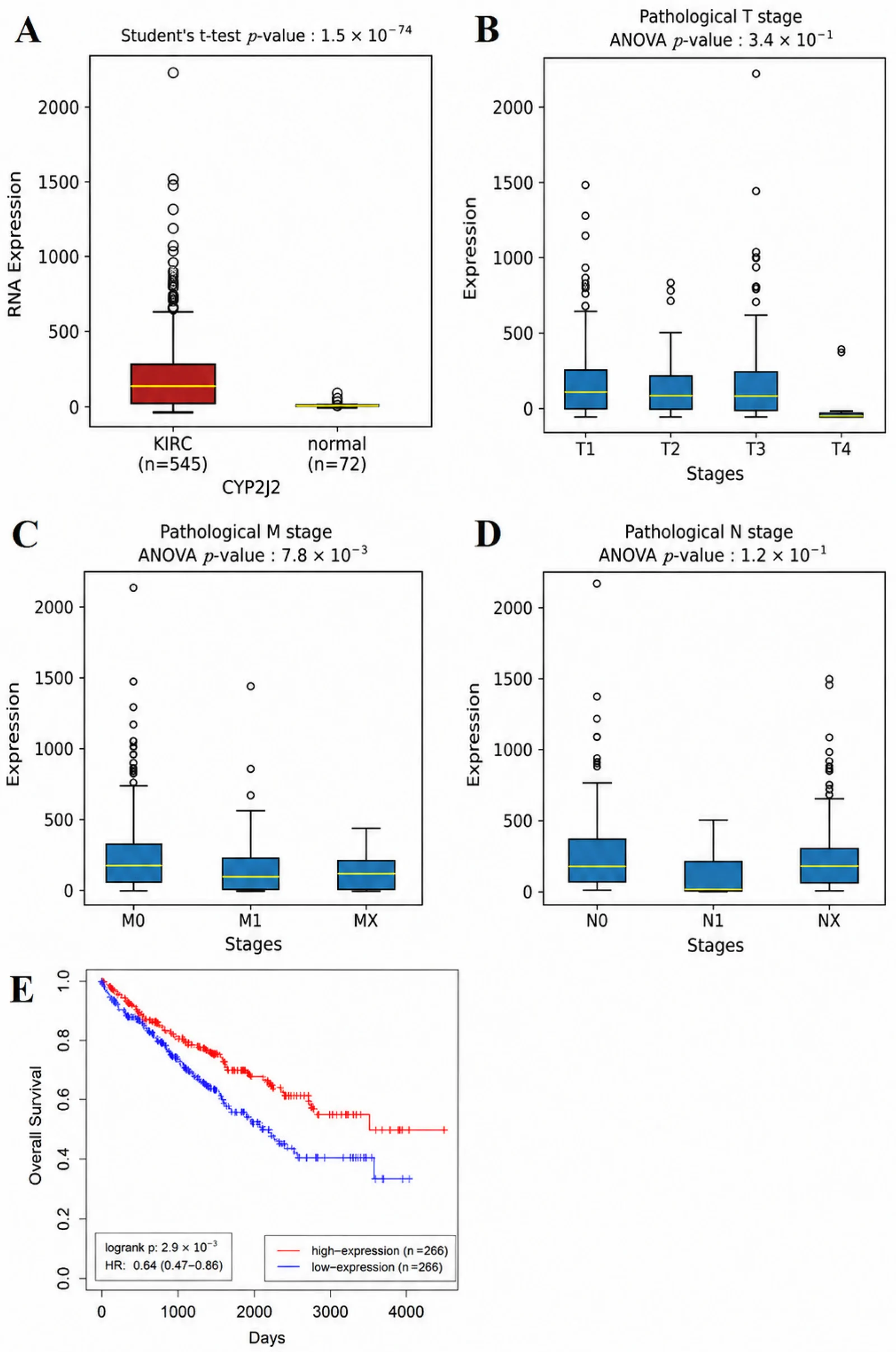
Transcriptomic analysis of CYP2J2 mRNA expression in clear cell renal cell carcinoma (ccRCC) using the TCGA-KIRC cohort. (**A**) Boxplot comparing CYP2J2 mRNA expression between ccRCC (KIRC; *n* = 545) and normal kidney tissues (*n* = 72). (**B**) Association between CYP2J2 mRNA expression and pathological T stage. (**C**) Association between CYP2J2 mRNA expression and pathological M stage. (**D**) Association between CYP2J2 mRNA expression and pathological N stage. (**E**) Kaplan–Meier overall survival analysis according to CYP2J2 mRNA expression.

Transcriptomic analysis revealed marked CYP2J2 mRNA overexpression in ccRCC compared with normal kidney tissue (Figure 4). The mean CYP2J2 expression was 232.6 in tumor tissues versus 7.0 in normal tissues (log2 fold change = 5.24). Interestingly, CYP2J2 expression was significantly associated with pathological M stage (*p* = 0.0079), with lower expression in tumors with distant metastasis (M1; mean = 181.6) and tumors with unclear metastatic status (MX; mean = 139.8) compared with non-metastatic tumors (M0; mean = 250.2). We observed no significant associations with pathological T stage (*p* = 0.34) or N stage (*p* = 0.12). Additionally, higher CYP2J2 mRNA expression was associated with longer overall survival (log-rank P = 0.0029; HR = 0.64, 95% CI: 0.47–0.86). Together, these findings suggest that although CYP2J2 transcription is markedly elevated in ccRCC compared with normal kidney tissue, higher expression is not associated with a more aggressive metastatic phenotype in this cohort. Because survival analysis was based on an unadjusted expression-group comparison and did not incorporate pathological stage or other potential prognostic covariates, this finding should be interpreted as an association with overall survival rather than evidence of an independent prognostic effect.

## 4. Discussion

This is the first study to assess CYP2J2 expression at both the mRNA and protein levels in prostate adenocarcinoma and ccRCC using transcriptomic and immunohistochemical analyses, respectively. The key results were that transcriptomic analysis revealed elevated CYP2J2 mRNA expression, especially in ccRCC, whereas CYP2J2 protein expression was very limited in both tumor types. Because transcriptomic alterations do not always correlate with protein levels, our results emphasize the need to assess both protein and transcript expression when evaluating potential tumor biomarkers. Modern proteogenomic research integrates transcriptomic and proteomic data to support biomarker validation [23,24].

Immunohistochemical analysis revealed limited CYP2J2 protein expression in the primary prostate adenocarcinoma and ccRCC cohorts, with positive staining in 7.0% and 1.0% of cases, respectively. This finding contrasts with an earlier investigation reporting CYP2J2 overexpression in various human tumors [11] and is consistent with evidence of variable tissue-specific CYP2J2 expression and function [25]. Moreover, we detected no statistically significant associations between CYP2J2 protein expression and the examined clinicopathological features. However, these analyses were substantially underpowered because of the low CYP2J2 positivity observed and the predominant TMA composition of early-stage and low-grade tumors, with only a few metastatic cases and a few high-stage or high-grade specimens. Therefore, the lack of statistically significant associations should be interpreted cautiously, as it does not exclude clinically relevant relationships that could not be detected in our cohorts.

On the other hand, transcriptomic analysis revealed elevated CYP2J2 mRNA expression in both tumor types, especially in ccRCC. Many CYP enzymes and several tumor biomarkers show similar differences in mRNA and protein expression. This discrepancy may indicate the involvement of several mechanistic processes, such as post-transcriptional regulation, protein turnover, or cellular heterogeneity within comprehensive transcriptome datasets; however, we did not explore these processes in the current study [23,26]. Additionally, the marked elevation of CYP2J2 mRNA along with the very limited protein detection observed in the separate IHC cohort further suggests that transcript abundance may not necessarily reflect CYP2J2 protein expression in ccRCC, reinforcing the possibility that tumor context and post-transcriptional regulation influence CYP2J2 expression [27,28]. Transcriptomic analysis also revealed a significant association between CYP2J2 expression and pathological T stage in prostate adenocarcinoma; however, this relationship was non-linear, with the highest expression in T2 tumors and the lowest expression in T4 tumors. Therefore, this finding does not support a simple relationship between higher CYP2J2 expression and more advanced prostate cancer. Similarly, higher CYP2J2 mRNA expression was associated with longer overall survival in ccRCC but not in prostate adenocarcinoma. However, these survival analyses were based on unadjusted Kaplan–Meier comparisons between expression-defined groups and did not account for pathological stage or other potential prognostic covariates. Therefore, the observed association between CYP2J2 mRNA expression and overall survival in ccRCC should not be interpreted as evidence of an independent prognostic effect. Importantly, these transcriptomic and survival analyses were derived from publicly available TCGA/OncoDB datasets and were not performed on the same patients as the immunohistochemical tissue microarrays. Therefore, these findings should be considered complementary evidence rather than direct patient-level validation of the protein findings and require confirmation in independent cohorts with paired molecular data [24,29,30,31].

Importantly, the findings in ccRCC suggest that CYP2J2′s biological role may be more context-dependent than a uniformly tumor-promoting function. Although CYP2J2 mRNA was markedly elevated in ccRCC compared with normal kidney tissue, its expression was lower in tumors with distant metastasis, and higher expression was associated with longer overall survival. This pattern contrasts with experimental studies reporting tumor-promoting effects of CYP2J2 in selected cancer models [11,12,13,14]. However, this is consistent with previous bioinformatic and transcriptomic studies showing elevated CYP2J2 expression and favorable survival associations in ccRCC [7,19]. Thus, increased CYP2J2 expression in ccRCC may reflect a tumor-specific metabolic or phenotypic state rather than necessarily indicating a driver of tumor progression. CYP2J2 expression may also be associated with a less aggressive or more differentiated renal phenotype. However, this remains a hypothesis because we did not directly assess differentiation markers and cellular composition in the present study. The lower expression in metastatic tumors and the favorable survival association therefore warrant further investigation using matched molecular data and functional studies to determine whether CYP2J2 contributes to, or merely reflects, a less aggressive ccRCC phenotype.

Several limitations of the present study should be acknowledged. The tissue microarrays for both tumor types used for immunohistochemical analysis may not fully reflect intratumoral heterogeneity. Furthermore, we performed no functional studies to investigate the mechanisms underlying altered CYP2J2 expression. Importantly, we generated the transcriptomic and survival analyses from publicly accessible TCGA/OncoDB datasets rather than from the same patient population as the immunohistochemical tissue microarrays. Consequently, the present study does not provide matched patient-level mRNA–protein validation, and the apparent discrepancy between CYP2J2 transcript and protein expression should be interpreted cautiously. Furthermore, the very low number of CYP2J2-positive tumors substantially limited the statistical power of the clinicopathological association analyses, particularly in the renal cohort. Therefore, the absence of statistically significant associations should be interpreted as reflecting an inability to reliably detect relationships rather than definitive evidence that such associations do not exist. Future studies using paired tumor specimens with matched transcriptomic and proteomic measurements, together with functional validation using appropriate in vitro and in vivo prostate and renal cancer models, are warranted to determine whether CYP2J2 plays a direct mechanistic role in tumor biology and to clarify its potential biological and clinical significance.

## 5. Conclusions

This study provides complementary immunohistochemical and transcriptomic characterization of CYP2J2 in prostate adenocarcinoma and ccRCC. CYP2J2 protein expression was infrequent with the IHC approach used, whereas analysis of public transcriptomic data revealed increased CYP2J2 mRNA expression, particularly in ccRCC. Although transcript expression showed associations with selected clinicopathological features and overall survival, these findings are exploratory and do not establish prognostic value, disease progression, or clinical utility. The observed difference between transcript and protein expression highlights the importance of evaluating CYP2J2 at multiple molecular levels. Further studies using larger independent cohorts, matched transcript–protein measurements, and appropriate in vitro and in vivo models are required to clarify the biological and potential clinical significance of CYP2J2 in prostate and renal cancers.

## Figures and Tables

**Table 1:** Association between CYP2J2 protein expression and clinicopathological characteristics of prostate adenocarcinoma specimens.

	CYP2J2 Expression		*p*-Value
	Negative (*n* = 93, 93.0%)	Positive (*n* = 7, 7.0%)	
Age			
≤65 years (*n* = 35, 35.0%)	31 (88.6%)	4 (11.4%)	0.236
>65 years (*n* = 65, 65.0%)	62 (95.4%)	3 (4.6%)	
Tumor depth of invasion			
T2 (*n* = 70, 70.0%)	66 (94.3%)	4 (5.7%)	0.239
T3 (*n* = 26, 26.0%)	24 (92.3%)	2 (7.7%)	
T4 (*n* = 4, 4.0%)	3 (75.0%)	1 (25.0%)	
Lymph node metastasis			
Negative (*n* = 85, 85.0%)	79 (92.9%)	6 (7.1%)	1.000
Positive (*n* = 15, 15.0%)	14 (93.3%)	1 (6.7%)	
Distant metastasis			
Negative (*n* = 94, 94.0%)	88 (93.6%)	6 (6.4%)	0.361
Positive (*n* = 6, 6.0%)	5 (83.3%)	1 (16.7%)	
Clinical stage			
Stage II (*n* = 51, 51.0%)	48 (94.1%)	3 (5.9%)	0.869
Stage III (*n* = 34, 34.0%)	31 (91.2%)	3 (8.8%)	
Stage IV (*n* = 15, 15.0%)	14 (93.3%)	1 (6.7%)	
Gleason grade			
Grade 1 (*n* = 20, 20.0%)	20 (100.0%)	0 (0.0%)	0.078
Grade 2 (*n* = 19, 19.0%)	16 (84.2%)	3 (15.8%)	
Grade 3 (*n* = 24, 24.0%)	24 (100.0%)	0 (0.0%)	
Grade 4 (*n* = 14, 14.0%)	12 (85.7%)	2 (14.3%)	
Grade 5 (*n* = 23, 23.0%)	21 (91.3%)	2 (8.7%)	

**Table 2:** Association between CYP2J2 protein expression and clinicopathological characteristics of renal tissue specimens.

	CYP2J2 Expression		*p*-Value
	Negative (*n* = 190, 99.0%)	Positive (*n* = 2, 1.0%)	
Sex			
Male (*n* = 132, 68.8%)	131 (99.2%)	1 (0.8%)	0.528
Female (*n* = 60, 31.3%)	59 (98.3%)	1 (1.7%)	
Age			
<65 years (*n* = 142, 74.0%)	140 (98.6%)	2 (1.4%)	1.000
≥65 years (*n* = 50, 26.0%)	50 (100.0%)	0 (0.0%)	
Tumor T stage			
T1 (*n* = 133, 63.9%)	132 (99.2%)	1 (0.8%)	0.521
T2 (*n* = 53, 27.6%)	52 (98.1%)	1 (1.9%)	
T3 (*n* = 5, 2.6%)	5 (100.0%)	0 (0.0%)	
T4 (*n* = 1, 0.5%)	1 (100.0%)	0 (0.0%)	
Lymph node metastasis			
Negative (*n* = 191, 99.5%)	189 (99.0%)	2 (1.0%)	1.000
Positive (*n* = 1, 0.5%)	1 (100.0%)	0 (0.0%)	
Distant metastasis			
Negative (*n* = 191, 99.5%)	189 (99.0%)	2 (1.0%)	1.000
Positive (*n* = 1, 0.5%)	1 (100.0%)	0 (0.0%)	
Clinical stage			
Stage I (*n* = 134, 69.8%)	133 (99.3%)	1 (0.7%)	0.514
Stage II (*n* = 52, 27.1%)	51 (98.1%)	1 (1.9%)	
Stage III (*n* = 5, 2.6%)	5 (100.0%)	0 (0.0%)	
Stage IV (*n* = 1, 0.5%)	1 (100.0%)	0 (0.0%)	
Histological grade			
Grade 1 (*n* = 173, 90.1%)	172 (99.4%)	1 (0.6%)	0.189
Grade 2 (*n* = 18, 9.4%)	17 (94.4%)	1 (5.6%)	
Grade 3 (*n* = 1, 0.5%)	1 (100.0%)	0 (0.0%)	

## Data Availability

The raw data supporting the findings of this study are provided in the Supplementary Materials. These include the raw Allred immunohistochemical scores, clinicopathological data used for the immunohistochemical analyses, and the exported OncoDB transcriptomic and survival analysis results.

## Supplementary Materials

The supporting information can be downloaded at: https://www.mdpi.com/article/10.3390/cancers18172809/s1, The following supplementary datasets are available with this article: Supplementary Table S1, raw CYP2J2 immunohistochemical and clinicopathological data for the prostate cohort; Supplementary Table S2, raw CYP2J2 immunohistochemical and clinicopathological data for the kidney cohort; Supplementary Table S3, CYP2J2 mRNA expression and survival data for the TCGA-PRAD cohort; and Supplementary Table S4, CYP2J2 mRNA expression and survival data for the TCGA-KIRC cohort.

## Abbreviations

The following abbreviations are used in this manuscript:

AJCC	American Joint Committee on Cancer
ccRCC	Clear cell renal cell carcinoma
CI	Confidence interval
CYP	Cytochrome P450
CYP2J2	Cytochrome P450 2J2
DAB	3,3′-Diaminobenzidine
EETs	Epoxyeicosatrienoic acids
FFPE	Formalin-fixed, paraffin-embedded
HR	Hazard ratio
IHC	Immunohistochemistry
KIRC	Kidney renal clear cell carcinoma
mRNA	Messenger RNA
PBS	Phosphate-buffered saline
PRAD	Prostate adenocarcinoma
RCC	Renal cell carcinoma
TCGA	The Cancer Genome Atlas
TMA	Tissue microarray
TNM	Tumor–node–metastasis
TPM	Transcripts per million
